# High frequency of vitamin D deficiency in current pregnant Japanese women associated with UV avoidance and hypo-vitamin D diet

**DOI:** 10.1371/journal.pone.0213264

**Published:** 2019-03-04

**Authors:** Kumiko T. Kanatani, Takeo Nakayama, Yuichi Adachi, Kei Hamazaki, Kazunari Onishi, Yukuo Konishi, Yasuyuki Kawanishi, Tohshin Go, Keiko Sato, Youichi Kurozawa, Hidekuni Inadera, Ikuo Konishi, Satoshi Sasaki, Hiroshi Oyama, Japan Environment and Children's Study Group

**Affiliations:** 1 Kyoto Regional Centre for Japan Environment and Children’s Study, Graduate School of Medicine, Kyoto University, Kyoto, Japan; 2 Department of Health Informatics, Kyoto University School of Public Health, Kyoto, Japan; 3 Department of Pediatrics, Faculty of Medicine, University of Toyama, Toyama, Japan; 4 Department of Public Health, University of Toyama, Toyama, Japan; 5 Department of Public Health, Tottori University, Tottori, Japan; 6 Doshisha University Center for Baby Science, Kyoto, Japan; 7 Institute for Advancement of Clinical and Translational Science, Kyoto University Hospital, Kyoto, Japan; 8 Department of Gynecology and Obstetrics, Kyoto University Graduate School of Medicine, Kyoto, Japan; 9 Department of Social and Preventive Epidemiology, School of Public Health, the University of Tokyo, Tokyo, Japan; 10 Department of Clinical Information Engineering Health Services Sciences, School of Public Health, Graduate School of Medicine, The University of Tokyo, Tokyo, Japan; Charles P. Darby Children's Research Institute, UNITED STATES

## Abstract

**Background:**

As a consequence of indoor occupations and reduced exposure to sunlight, concerns have been raised that vitamin D deficiency is widespread in developed countries. Vitamin D is known to be associated with increased risks of morbidity and mortality in various diseases.

**Objective:**

To investigate the serum vitamin D status and its relation with life-style factors in pregnant Japanese women.

**Methods:**

Among a cohort for 3,327 pregnant women who participated in an the adjunct study of the Japan Environment and Children's Study during 2011–2013, in which data were obtained on various life-style factors, including both dietary intake of vitamin D and frequency of UV exposure, this study consisted of 1,592 pregnant women, from whom 2,030 serum samples were drawn in Jan, Apr, Jul, and Oct, and the association between serum 25(OH)D level and life-style factors were analyzed using linear mixed models.

**Results:**

Serum 25(OH)D levels were less than 20ng/mL in 1,486 of 2,030 samples (73.2%). There was an obvious seasonal change, with serum 25(OH)D levels of less than 20 ng/mL in 89.8% and 47.8% of samples in spring (April) and autumn (October), respectively. Both the frequency spent under sunlight and dietary intake of vitamin D were significantly associated with serum 25(OH)D level. An increase in sunlight exposure of more than 15 min for 1 to 2 days per week in non-winter, or dietary intake of 2 μg/day of vitamin D resulted in an elevation of 1 ng/mL in serum 25(OH)D levels.

**Conclusion:**

These findings indicate that vitamin D deficiency is very severe in Japanese pregnant women, especially those rarely exposed to sunlight. The benefits of UV rays should also be informed of when its risk is alerted, and clinicians should propose the adequate UV exposure level.

## Introduction

Vitamin D is a fat-soluble secosteroid with well-established effects on calcium homeostasis. More recently, vitamin D has also been recognized to interact with a nuclear receptor in various other organs[1] and its deficiency is associated with increased risks of morbidity and mortality in various diseases including cardiovascular, malignant, and autoimmune diseases[2,3]. Accumulating evidence suggests that vitamin D deficiency during pregnancy may cause complications such as preeclampsia[3–5], although its implications and the underlying mechanisms are not fully understood. And it is even hypothesized that vitamin D deficiency in the fetal period leads to an increased risk of allergic diseases, multiple sclerosis, and cardiovascular diseases in later life[2,6–8].

As a consequence of indoor occupations and reduced exposure to sunlight, concerns have been raised that vitamin D deficiency is widespread in developed countries[2,3]. In Japan, because fish is a primary component of the traditional diet, the risk of vitamin D deficiency is rarely discussed. However, studies indicate that younger people consume less fish [9–12], and while females appear to be at higher risk of vitamin D deficiency because they tend to avoid direct sunlight exposure to prevent skin-tanning, may be malnourished from maintaining a lean proportion [13]. The importance of monitoring the vitamin D status in Japan has only recently been demonstrated [14,15].

Accordingly, the present study aimed to examine the serum vitamin D status of pregnant Japanese women and to estimate the impact of lifestyle factors on vitamin D levels in a population-based cohort.

## Methods

### Study design

This was cross-sectional sub-study comprising pregnant Japanese women enrolled in the adjunct study of the Japan Environment and Children's Study (JECS) to examine the effects of desert dust exposure on allergic diseases in pregnant women and their children in three areas in Japan; Kyoto (N 35°), Toyama (N 36°), and Tottori (N 35.5°) [16]. The study protocol was approved by ethic comities in Kyoto University, University of Toyama, and Tottori University, and was registered at UMIN000010826[16].

The details of the study design and protocol have been previously reported [16]. In brief, the JECS is a community based national birth cohort study[17,18], and the JECS participants from the above three regions who agreed to participate in the adjunct study were enrolled prior to delivery. Questionnaires on lifestyle factors and diet were sent out twice through the JECS[17,18]. Serum samples were taken three times during pregnancy; during the first trimester, during the second trimester, and at the timing of delivery[17,18].

Serum 25(OH)D levels were measured in blood samples from Jan (winter), April (spring), July (summer) and October (autumn) to evaluate their vitamin D distributions and seasonal changes, and the impact of lifestyle and dietary factors on 25(OH)D levels were estimated.

### Measurements

#### Demographics

Information on various demographic parameters, including age, pre-pregnancy BMI, housing environment, socioeconomic background, smoking habit, and history of allergic and other diseases, was obtained from the majority of the study population during the 1^st^ trimesters of pregnancy. Full details of demographic parameters are described elsewhere[17].

#### Serum vitamin D levels

Serum samples were stored at -30°C until biochemical analysis of blood was performed on serum samples at SRL laboratories (Tokyo, Japan). 25(OH)D was measured using the 25(OH)D 125I RIA kit (DiaSorin Inc, Minnesota, USA) [19,20]. The measuring range was 6 to 99900000 ng/ml. Measurement values below the limit of quantification (LOQ) were assigned 50% of the LOQ. Serum 25(OH)D concentrations were natural log-transformed before statistical tests were performed.

#### Estimated dietary vitamin D intake

A validated self-administered Food Frequency Questionnaire (FFQ) [21–23] was administered twice during pregnancy. The first FFQ was used for the main analysis, and the second FFQ for sensitivity analysis. Vitamin D intake was adjusted by the total energy using the energy-density method[24]. We excluded subjects who returned unreasonable range of total energy (less than 50% or more than 150% of predicted values) for the main analysis and we confirmed the robustness of the result by further analysis including the data from excluded subjects.

#### Estimated vitamin D supplementation

Dietary vitamin D supplementation was evaluated by a self-administered questionnaire on the frequency and the bland name of vitamin supplements. The dose of vitamin D within each reported tablet was searched, and the subject was deemed as ‘supplemented’ if any dose of vitamin D was contained in the tablet.

#### UV exposure frequency

A self-administered questionnaire was sent to the subject via their mobile phones [16]. Questions included “On a typical day, how often are you exposed to sunlight for more than 15 minutes from 9 am to 3 pm? Please include the time spent exposed to sunlight under trees or under light clouds.—More than 5 days a week—3–4 days a week—1–2 days a week—rarely”, “How often, for leisure purposes only, are you exposed to sunlight for more than 15 minutes from 9 am to 3 pm? Please include the time spent exposed to sunlight under trees or under light clouds.—Almost weekly—2–3 times a month—once a month—rarely”, and “On a typical day, do you protect your hands and neck from UV rays?—Never expose bare skin under direct sunlight, even in winter—Often block UV rays with cream or sunshades in seasons with strong UV rays—Seldom protect against UV rays”.

#### Other factors

The following potential influencing factors were examined: age, pre-pregnancy BMI, pregnancy trimester, past history of allergic diseases (asthma, allergic rhinitis, and atopic dermatitis), skin type according to self reported reaction to UV light exposure, how important they think of body weight control during pregnancy, intentional avoidance of fish or eggs, employment in agriculture or fishery, frequency of night-shift working, smoking habit, family income and education level. Age and pre-pregnancy BMI were obtained from the physicians’ record, and others were obtained via a self-administered questionnaire[16–18].

### Statistical analysis

Average 25(OH)D levels among groups were estimated using the linear mixed model analysis, with intra-individual variation by repeated measurements accounted for. For comparison of variables among more than two groups, p values were adjusted by Dunnett’s method.

A uni-variate model was applied for each factor, followed by multi-variate analysis incorporating all variables with p values of <0.1 in the univariate models. Backward elimination method was applied to construct the final model.

**Fig 1:**
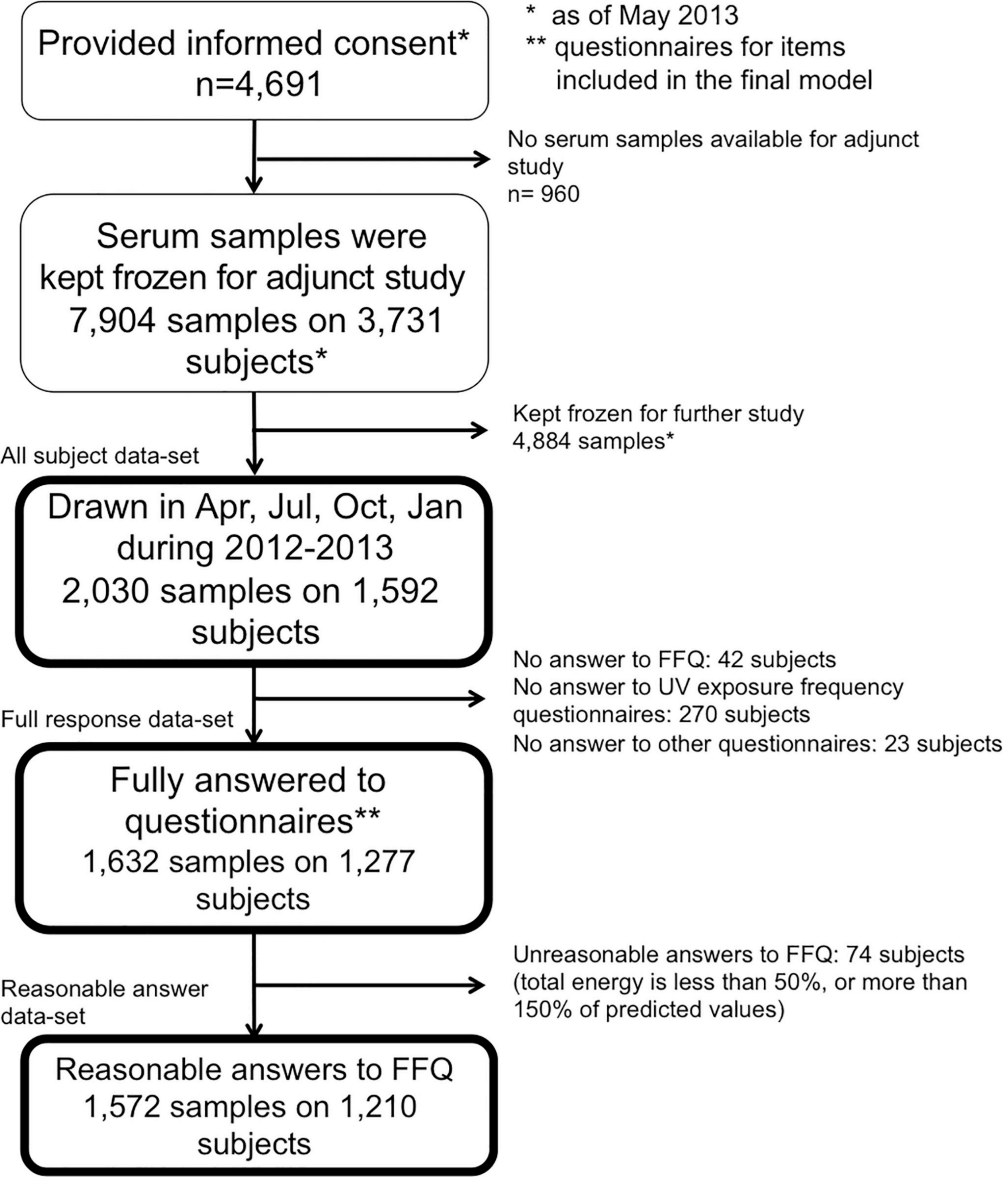
Serum samples analyzed in the sub-study of the Japan Environment and Children’s Study (JECS).

The entire cohort dataset (Fig 1) was used for analysis of seasonal 25(OH)D changes. The reasonable answer dataset, which excluded subjects with total calories on the FFQ of less than 50% and more than 150% of predicted values (Fig 1) was used for the analysis that include dietary intake of vitamin D, and the full answer dataset (Fig 1) was used for sensitivity analysis.

All analyses were performed using SAS software, version 9.3 (SAS Institute), and two-sided P<0.05 were considered statistically significant.

## Results

### Subjects

Of the 6,340 serum samples from 3,495 pregnant women who participated before May 2013 in the adjunct study, 2,030 samples which were collected in Apr, Jul, Oct, and Jan during 2012 to 2013 from 1,592 pregnant women, were included in this sub-study, as illustrated in Fig 1.

Table 1 summarizes the characteristics of the study cohort. All subjects were pregnant with an age range from teenagers to over 45 years, and various socioeconomic backgrounds. Subject characteristics were similar to those reported by the Japanese government in 2012, except that the proportion of current smokers was lower in the study cohort (1.6–2.7%) than that in the government report (12.8% for women in their 20s and 16.6% for women in their 30s) [25,26]. The proportion of subjects with an education level up to junior high school completion was lower (1.6–3.3%) compared with the government report (6.0%)[27,28]. Overall, the study cohort was considered to be a good representation of pregnant women in Japan.

**Table 1:** Characteristics of study cohort.

	Entire cohort dataset n = 1,592	Reasonable answer dataset n = 1,210
**Age (years) ^a^^b^**	31.4±4.7	31.7±4.6
**Height (cm) ^a^^b^**	158.4±5.3	158.6±5.3
**BMI before pregnancy ^a^^b^**	20.8±3.2	20.9±2.8
**Dietary vitamin D intake (μg/day) ^a^^b^**	5.5±2.8	5.3±2.8 ^**c**^
**Use of vitamin D supplements ^b^**	n (%)	n (%)
Yes	79 (5.0)	62 (5.1)
No	1465 (92.0)	1148 (94.9)
No response ^d^	48 (3.0)	0 (0)
**Agree with the importance of BW control**	n (%)	n (%)
**during pregnancy? ^b^** Totally agree	828 (52.0)	649 (53.6)
Agree	631 (39.6)	495 (40.9)
Cannot decide	70 (4.4)	55 (4.6)
Disagree	10 (0.6)	8 (0.8)
Totally disagree	2 (0.1)	2 (0.2)
No response ^d^	51 (3.2)	1 (0.1)
**Intentionally did not eat eggs**	n (%)	n (%)
**during pregnancy ^b^** Yes	26 (1.6)	20 (1.7)
No	1518 (95.4)	1190 (98.4)
No response ^d^	48 (3.0)	0 (0)
**Intentionally did not eat fish**	n (%)	n (%)
**during pregnancy ^b^** Yes	36 (2.3)	26 (2.2)
No	1508 (94.7)	1184 (97.9)
No response ^d^	48 (3.0)	0 (0)
**Usage of sunscreen on neck and hands**	n (%)	n (%)
Always	36 (2.3)	33 (2.7)
Only in summer	767 (48.2)	706 (58.4)
Rarely	466 (29.3)	422 (34.9)
No response ^d^	323 (20.3)	49 (4.1)
**Frequency of UV exposure in daily life**	n (%)	n (%)
Rarely	239 (15.0)	216 (17.9)
Once to twice a week	343 (21.6)	314 (26.0)
Three to four times a week	333 (20.9)	309 (25.5)
More than 5 times a week	407 (25.6)	371 (30.7)
No response ^d^	270 (17.0)	0 (0)
**Frequency of UV exposure at weekends**	n (%)	n (%)
Rarely	242 (15.2)	218 (18.0)
Once a month	57 (3.6)	53 (4.4)
Twice to three times a month	341 (21.4)	315 (26.0)
Every weekend	659 (41.4)	601 (49.7)
No response ^d^	293 (18.4)	23 (1.9)
**Self-reported skin reaction to UV light**	n (%)	n (%)
Burns easily, never tans	109 (6.9)	93 (7.7)
Burns easily, tans minimally with difficulty	521 (32.7)	472 (39.0)
Burns moderately, tans moderately	465 (29.2)	431 (35.6)
Burns minimally, tans moderately and easily	122 (7.7)	110 (9.1)
Rarely burns, tans profusely	6 (0.4)	5 (0.4)
No response ^d^	369 (23.2)	99 (8.2)
**History of allergic diseases**	n (%)	n (%)
Asthma	182 (11.4)	143 (11.8)
Allergic rhinitis	631 (39.6)	504 (41.7)
Atopic dermatitis	273 (17.2)	222 (18.4)
**Works in fishery or agriculture ^b^**	n (%)	n (%)
Yes	6 (0.4)	4 (0.3)
No	1521 (95.5)	1193 (98.6)
No response ^d^	65 (4.1)	13 (1.1)
**Night-shift ^b^**	n (%)	n (%)
Never	1400 (87.9)	1105 (91.3)
One to two times a month	84 (5.3)	61 (5.0)
Three to four times a week	52 (3.3)	40 (3.3)
More than five times a week	5 (0.3)	2 (0.2)
No response ^d^	51 (3.2)	2 (0.2)
**Smoking history ^b^**	n (%)	n (%)
Never smoked	1009 (65.0)	784 (64.8)
Stopped before pregnancy	382 (24.0)	299 (24.7)
Stopped during pregnancy	138 (8.7)	100 (8.3)
Current smoker	37 (2.3)	24 (2.0)
No response ^d^	26 (1.6)	3 (0.3)
**Partner’s smoking history ^b^**	n (%)	n (%)
Never smoked	508 (31.9)	394 (32.6)
Stopped before pregnancy	409 (25.7)	320 (26.5)
Stopped after pregnancy	28 (1.8)	17 (1.4)
Current smoker	608 (38.2)	467 (38.6)
No response ^d^	39 (2.5)	12 (1.0)
**Education level ^b^**	n (%)	n (%)
Junior high school	42 (2.6)	31 (2.6)
High school	369 (23.2)	268 (22.2)
Vocational school	330 (20.7)	227 (18.8)
College (2 years)	311 (19.5)	246 (20.3)
High vocational School	21 (1.3)	17 (1.4)
College (4 years)	453 (28.5)	382 (31.6)
Graduate school	39 (2.5)	36 (3.0)
No response ^d^	27 (1.7)	3 (0.3)
**Family annual income ^b^**	n (%)	n (%)
Under $20,000	51 (3.2)	30 (2.5)
$20,000–40,000	433 (27.2)	328 (27.1)
$40,000–60,000	530 (33.3)	417 (34.5)
$60,000–80,000	271 (17.0)	220 (18.2)
$80,000–100,000	111 (7.0)	93 (7.7)
Above $100,000	82 (5.2)	64 (5.3)
No response ^d^	114 (7.2)	58 (4.8)

^a^ Values are expressed as the mean ± SD.

^b^ Data from Japan Environment and Children's Study (JECS).

^c^ Adjusted by total energy.

^d^ No response corresponds to those who did not return the questionnaire.

### Frequency of UV exposure and vitamin D status

**Fig 2:**
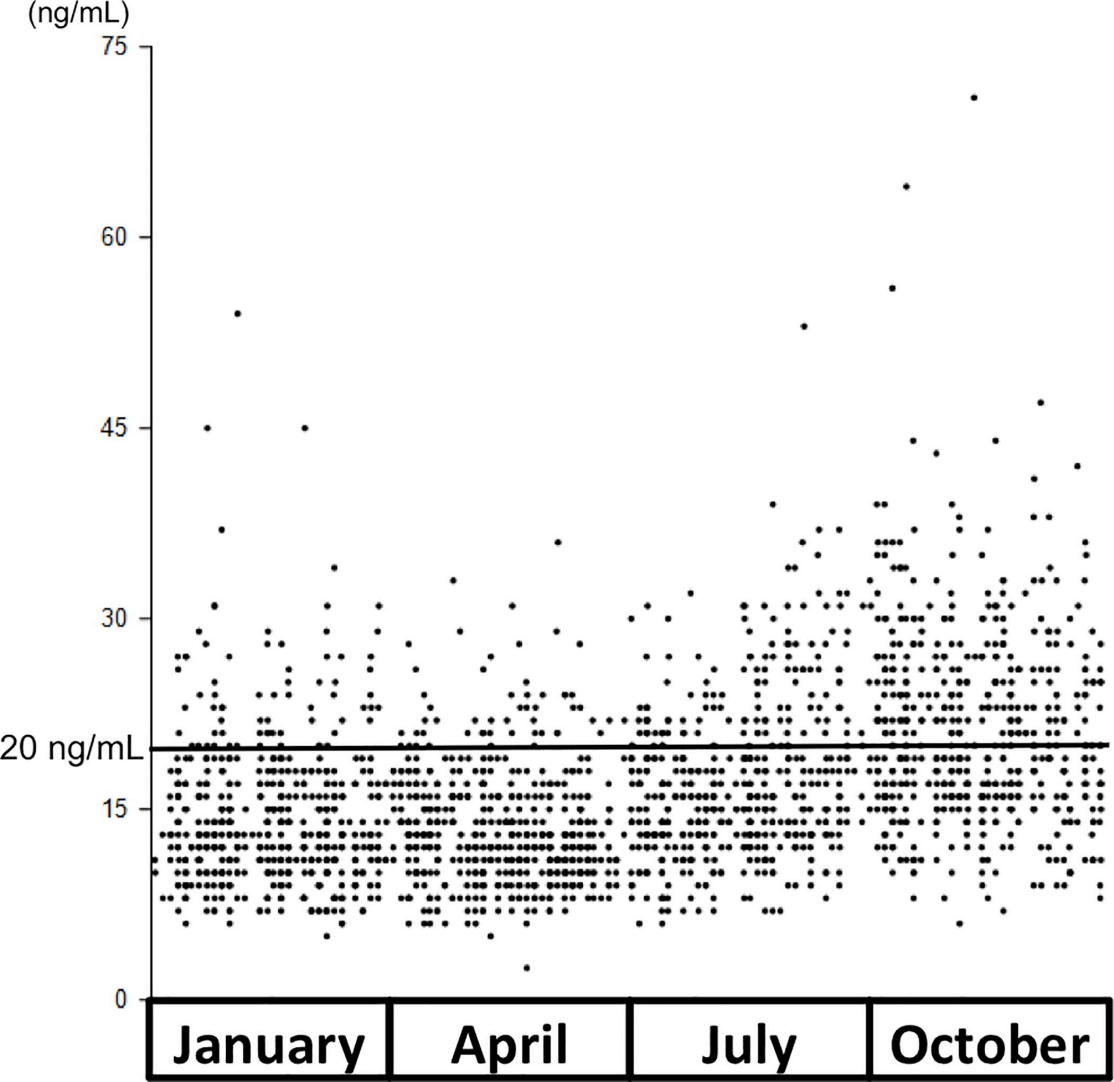
Seasonal changes in serum 25(OH)D levels.

**Fig 3:**
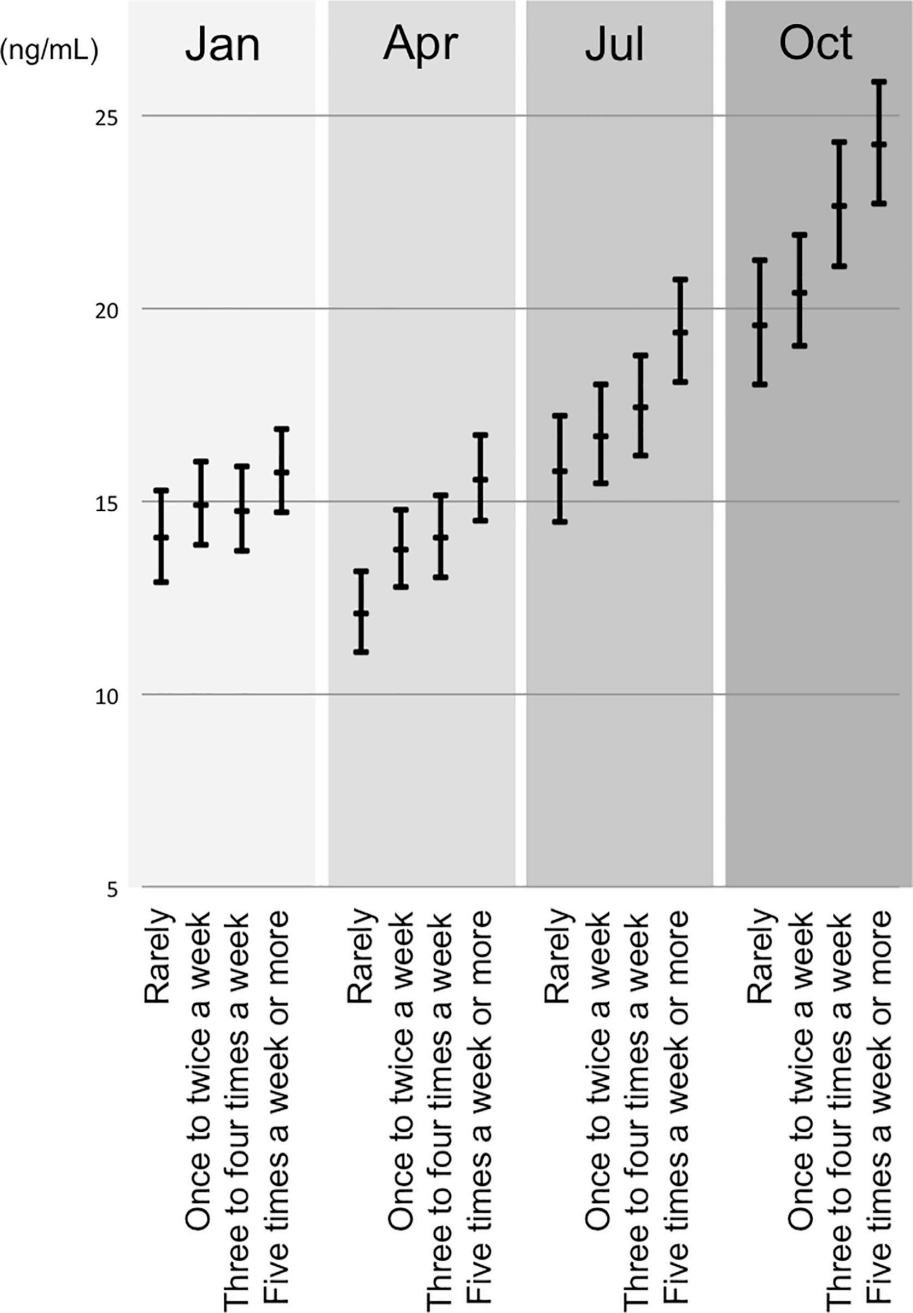
Serum 25(OH)D levels during each month in relation to the frequency of sunlight exposure. Linear mixed model with random effect of repeated measurements. Adjusted by location, dietary vitamin D intake, dietary calorie intake, vitamin D supplementation, pregnancy trimester, and if they live with children.

The mean serum 25(OH)D level was 16.7± 6.99 ng/mL (range: <5–71 ng/mL). Vitamin D deficiency, defined as less than 20ng/mL, was present in 73.2% (1,486 of 2,030 samples), and 10.8% (219 of 2,030 samples) had less than 10 ng/mL, which is defined as severe vitamin D deficiency. The distribution showed a clear seasonal change (Fig 2, Table 2), and 87.7% (880 of 1,003 samples) had less than 20ng/mL in winter and spring. This trend was observed even among women who reported sunlight exposure for at least 15 minutes on more than five days a week (Fig 3). However, at the end of summer (October), the mean 25(OH)D level of the group was much higher compared with that of subjects with least exposed to sunlight (Fig 3), and 61.5% (88 of 143 samples) of subjects who reported sunlight exposure for at least 15 minutes on more than five days a week achieved 20 ng/mL, while 34.6% (27 of 78 samples) of subjects with least exposed to sunlight achieved 20 ng/mL.

**Table 2:** Serum 25(OH) levels (least square means; LS means and 95% confidence intervals; 95%CI) in univariate models.

	LS means	95% CI			Adjusted P value
**Season**						
Winter (Jan)	13.5	13.1	−	13.9	ref
Spring (Apr)	12.7	12.3	−	13.1	.006
Summer (Jul)	16.3	15.9	−	16.8	< .0001
Autumn (Oct)	20.2	19.6	−	20.7	< .0001
**Residential location**					
Kyoto (N35)	15.1	14.6	−	15.6	ref
Toyama (N36)	16.1	15.7	−	16.5	.008
Tottori (N35.5)	14.4	13.8	−	15.0	.105
**Frequency of UV exposure in daily life**					< .0001 ^a^
Rarely	13.8	13.2	−	14.4	ref
Once a week	14.8	14.2	−	15.4	.062
Two to three times a week	15.5	14.9	−	16.1	.001
More than four times a week	16.8	16.2	−	17.4	< .0001
**Frequency of UV exposure at weekends**					< .0001 ^a^
Rarely	14.0	13.3	−	14.6	ref
Once a month	15.7	14.3	−	17.3	.082
Two to three times a month	15.1	14.5	−	15.7	.038
Every week	16.0	15.6	−	16.5	< .0001
**Usage of sunscreen on neck and hands**					.188 ^a^
Always	13.9	12.3	−	15.8	ref
Only in summer	15.3	14.9	−	15.7	.186
Rarely	15.6	15.1	−	16.1	.106
**Self-reported skin reaction to UV exposure**					.293^a^
Burns easily, never tans	14.5	13.5	−	15.6	.203
Burns easily, tans minimally with difficulty	15.7	15.2	−	16.2	ref
Burns moderately, tans moderately	15.2	14.7	−	15.7	.996
Burns minimally, tans moderately and easily	15.8	14.8	−	16.9	.999
Rarely burns, tans profusely	15.9	11.9	−	21.2	.057
**Dietary vitamin D intake ^b^^b^**					< .0001 ^a^
1^st^ quartile (–3.8μg/day)	14.6	14.0	−	15.1	ref
2^nd^ quartile (3.8–5.2μg/day)	15.0	14.5	−	15.6	.486
3^rd^ quartile (5.2–6.9μg/day)	15.7	15.1	−	16.3	.019
4^th^ quartile (6.9 –μg/day)	17.0	16.4	−	17.6	< .0001
**Use of vitamin D supplements**					
No	15.3	15.0	−	15.6	ref
Yes	19.4	17.9	−	21.0	< .0001
**Pregnant trimester**					
1^st^ trimester	14.5	14.0	−	15.1	< .0001
2^nd^ trimester	16.2	15.8	−	16.7	ref
At delivery	15.0	14.6	−	15.4	< .0001
**Age**					.032 ^a^
<25 years	14.1	13.1	−	15.1	.032
25–35 years	15.4	15.1	−	16.2	ref
>35 years	15.7	15.1	−	15.8	.759
**BMI before pregnancy**					.519
<18.5	15.1	14.4	−	15.7	.439
18.5–25	15.5	15.2	−	15.8	ref
>25	15.4	14.5	−	16.4	.974
**History of asthma**					
No	15.4	15.1	−	15.7	ref
Yes	15.4	14.6	−	16.3	.974
**History of allergic rhinitis**					
No	15.4	15.1	−	15.8	ref
Yes	15.4	14.9	−	15.8	.860
**History of atopic dermatitis**					
No	15.4	15.1	−	15.7	ref
Yes	15.4	14.7	−	16.1	.905
**Works in fishery or agriculture**					
Yes	18.9	14.0	−	25.5	.198
No	15.5	15.2	−	15.8	ref
**Night-shift**					.428 ^a^
Never	15.6	15.3	−	15.9	.514
One to three times a month	14.8	13.7	−	16.0	ref
One to four times a week	14.7	13.3	−	16.3	.999
More than five times a week	17.1	12.1	−	24.1	.782
**Family income**					.1194 ^a^
Under $20,000	15.0	13.7	−	16.5	.9996
$20,000–40,000	15.2	14.7	−	15.7	ref
$40,000–60,000	15.7	15.3	−	16.2	.5378
$60,000–80,000	15.6	15.0	−	16.3	.8372
$80,000–100,000	15.6	14.6	−	16.6	.9588
Above $100,000	16.0	14.9	−	17.2	.6913
**Smoking status**					.015 ^a^
Never smoked	15.6	15.3	−	16.0	ref
Stopped before pregnancy	15.6	15.1	−	16.2	1.00
Stopped during pregnancy	14.3	13.4	−	15.2	.018
Current smoker	14.0	12.5	−	15.8	.204
**Smoking status of subjects’ partners**					.019 ^a^
Never smoked	15.7	15.2	−	16.2	ref
Stopped before pregnancy	15.8	15.3	−	16.4	.971
Stopped after pregnancy	17.0	14.8	−	19.6	.567
Current smoker	14.9	14.5	−	15.4	.068

Entire cohort dataset (excluding non-responders for each question).

All values are from univariate linear mixed models, with 25(OH)D natural log-transformed.

LS means and 95%CIs are shown as exponentials of log-transformed 25(OH)D.

For variables with more than three groups, p values are adjusted by Dunnett.

^a^ P values for trend.

^b^ Reasonable answer dataset, total energy adjusted by residual method.

Furthermore, vitamin D levels were evaluated in subjects employed in agriculture/fishery, who work outside during their daily lives. As expected, this group showed higher 25(OH)D levels, especially in autumn (LS mean 33.6 ng/mL, 95%CI; 17.8–63.6), although the number of subjects employed in agriculture/fishery was very few (7 samples on 6 subjects) and a statistical significance was not achieved.

Unexpectedly, the subjects whose self-reported skin type was fair tended to have a lower 25(OH)D levels than other skin types in this relatively homogenous population (Table 2).

An increase in the frequency of sunlight exposure to at least 15 minutes for 1 to 2 days per week resulted in elevations in 25(OH)D levels of approximately 1 ng/mL in non-winter and 0.5 ng/mL in winter (Fig 3).

### Vitamin D status and dietary vitamin D intake

The mean dietary intake of vitamin D (energy-adjusted) in Japanese pregnant women was estimated to be 5.5±2.8 **μ**g/day. As few as 22.6% of subjects consumed above 7.0 **μ**g/day, described as the “adequate intake” per day for Japanese pregnant women[29]. Nobody exceeded 100**μ**g/day, described as the “tolerable upper intake” per day in the guideline.

**Fig 4:**
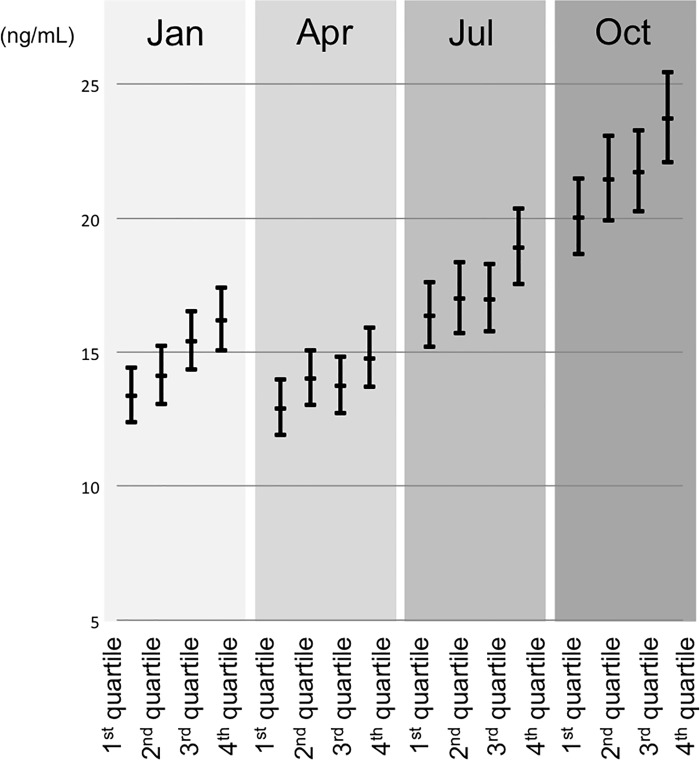
Serum 25(OH)D levels during each month in relation to the daily dietary intake of vitamin D. Linear mixed model with random effect of repeated measurements. Adjusted by location, frequency of sunlight exposure, dietary calorie intake, vitamin D supplementation, pregnancy trimester, and if they live with children. Average of 1^st^ quartile was 2.5**μ**g/day, and average of 4^th^ quartile was 9.4**μ**g/day.

The amount of dietary vitamin D intake was significantly associated with the 25(OH)D level (Tables 2 and 3, Fig 4), and 198 (90.8%) of 218 samples from subjects with above 7.0 **μ**g/day (energy-adjusted) had above 10 ng/mL even in winter and spring, although the majority (174/218, 79.8%) of these subjects did not achieve 20 ng/mL. The average daily intake of vitamin D in women with above 20 ng/mL of 25(OH)D in winter and spring was 6.5**μ**g/day.

**Table 3:** Serum 25(OH) levels (least square means; LS means and 95% confidence intervals; 95%CI) in multivariate models.

	LS means	95%CI			p value
**Residential location**					< .001
Kyoto (N35)	16.4	15.6	-	17.2	ref
Toyama (N36)	17.7	16.8	-	18.4	.002
Tottori (N35.5)	16.0	15.1	-	16.9	.570
**Season**					< .001
Winter (Jan)	14.8	14.1	-	15.5	ref
Spring (Apr)	13.8	13.2	-	14.6	.009
Summer (Jul)	17.3	16.4	-	18.2	< .0001
Autumn (Oct)	21.7	20.6	-	22.7	< .0001
**Pregnancy trimester**					< .0001
1^st^ trimester	16.1	15.3	-	17.0	< .0001
2^nd^ trimester	17.7	17.0	-	18.6	Ref
At delivery	16.1	15.4	-	16.8	< .0001
**Use of vitamin D supplements**					
No	14.7	14.4	-	15.0	Ref
Yes	19.0	17.5	-	20.5	< .0001
**Dietary vitamin D intake (μg/day) ^a^**					< .0001
1^st^ quartile (– 3.8μg/day)	15.4	14.6	-	16.3	ref
2^nd^ quartile (3.8–5.2μg/day)	16.4	15.5	-	17.3	.052
3^rd^ quartile (5.2–6.9μg/day)	16.7	15.9	-	17.6	.006
4^th^ quartile (6.9 – μg/day)	18.1	17.2	-	19.1	< .0001
**Frequency of UV exposure in daily life**					< .0001
Rarely	15.1	14.3	-	16.0	ref
Once a week	16.2	15.4	-	17.1	.017
Two to three times a week	16.9	16.1	-	17.9	.0001
More than four times a week	18.4	17.5	-	19.4	< .0001

Reasonable answer dataset

Multivariate linear mixed models, with 25(OH)D natural log-transformed.

LS means and 95%CIs are shown as exponentials of natural log-transformed 25(OH)D.

For variables with more than three groups, the p values are adjusted by Dunnett.

^a^ Adjusted by total energy by residual method.

An increase of 1**μ**g/day dietary vitamin D intake led to an elevation of approximately 0.5 ng/mL in serum 25(OH)D (Fig 4).

### Other factors associated with 25(OH)D levels

In the univariate linear mixed models with random effect of repeated measurements, the following factors were found to be significantly associated with 25(OH)D level (Table 2); residential location, frequency of UV exposure in daily life, frequency of UV exposure at weekends, month of blood sample collection, pregnancy trimester of blood sample, use of vitamin D supplements, dietary intake of vitamin D, dietary calorie intake, living with children, and smoking habits of subjects and their partners.

In the multivariate model incorporating all these variables, the following were consecutively excluded; frequency of UV exposure at weekends, age, smoking of partner, and smoking. The final model included the following factors; month of blood collection, residential location, pregnancy trimester of blood collection, use of vitamin D supplements, frequency of UV exposure in daily life, and dietary intake of vitamin D (Table 3).

Vitamin D supplementation, multivitamin tablets or calcium tablets, was reported by very few pregnant women (5.1%). Most tablets contained 2.5–5.0 **μ**g/day of vitamin D, and 15**μ**g/day at most (one case). However, their serum increase of 25(OH)D level were as much as 4.5 ng/mL (Table 3).

Contrary to previous reports from other countries, a univariate model showed that age was negatively associated with 25(OH)D levels in Japanese pregnant women (Table 2). This tendency was lost in the multivariate model or a model including covariates of dietary intake of vitamin D, UV exposure frequency, and living with children. Therefore it is suggested that this was the consequence of confounding effects of less exposure to sunlight and lower dietary consumption of vitamin D among younger populations in Japan[9,30].

**Fig 5:**
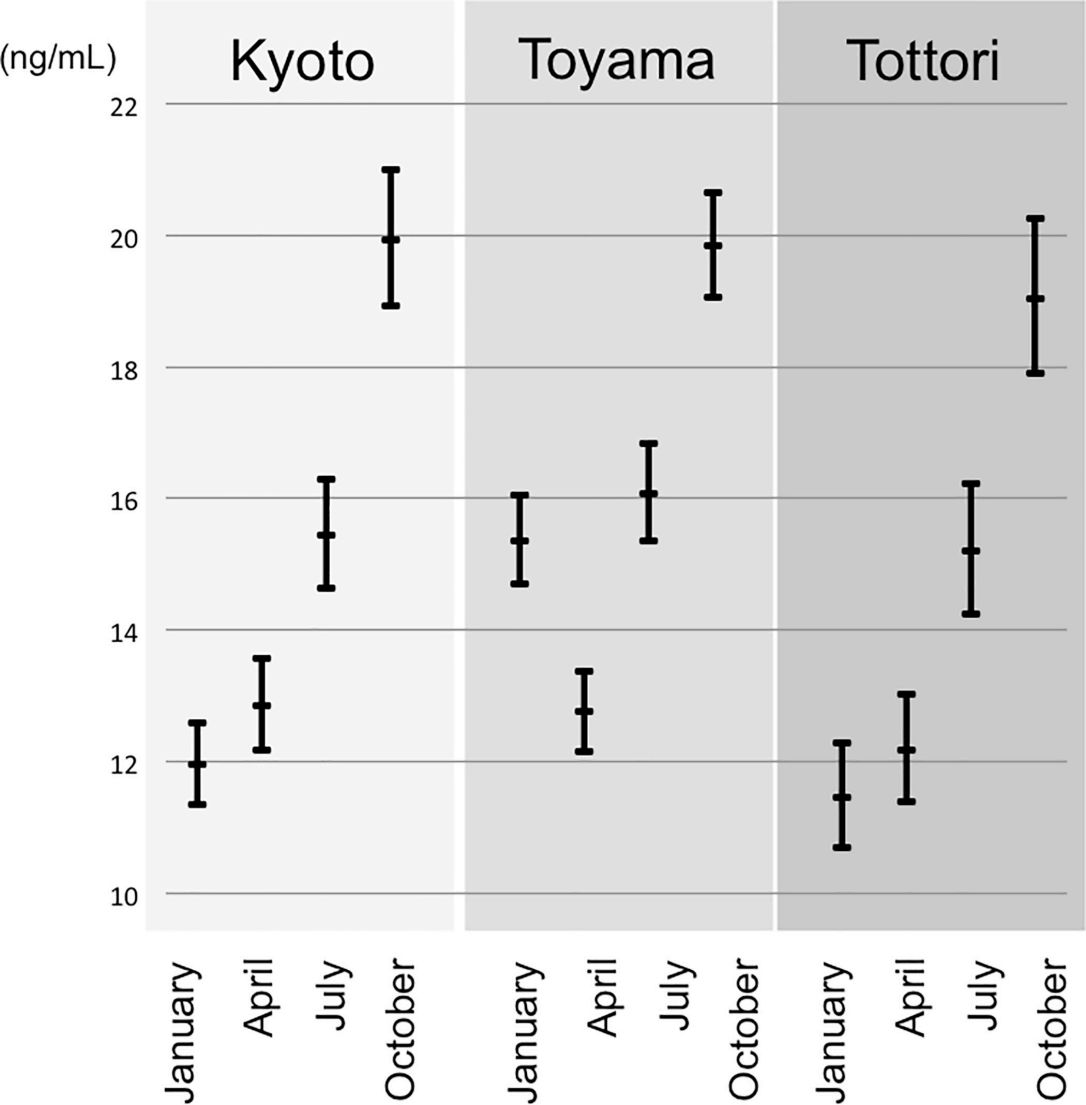
Serum 25(OH)D levels in Kyoto, Toyama, and Tottori in relation to the sampled months. Linear mixed model with random effect of repeated measurements. Adjusted by location, frequency of sunlight exposure, dietary vitamin D intake, dietary calorie intake, vitamin D supplementation, pregnancy trimester, and if they live with children.

Unexpectedly, even after the adjustment for lifestyle and dietary variables, pregnant women living in Toyama, the northern-most of the three locations, exhibited significantly higher 25(OH)D levels than in the other two locations (Table 3). By examining other characteristics, it was found that only samples taken in winter (January) in Toyama showed higher 25(OH)D levels than in the other two locations (Fig 5). Because Toyama is famous for cold yellowtail and other seafood products in winter, these women may have had a fish-rich diet in winter, although this was not sufficiently reflected in the answers to the questionnaire on “typical” diet. Furthermore, Toyama had higher snowfall than the other two locations in January 2013 (Toyama: 4cm/day, Tottori: 0.8cm/day, Kyoto: 0cm/day) and some sunlight hours (Toyama: 79 hours /month, Tottori 73 hours /month, Kyoto: 124 hours /month). Reflection of UV rays from snow on the ground may have increased UV exposures. This may be a strategy for maintaining adequate serum 25(OH)D levels for people living in northern, snowy areas, and should be confirmed by further studies.

## Discussion

In this study, we showed that Japanese pregnant women are in severe vitamin D deficiency status (10.8% are <10 ng/mL, 73.2% are <20 ng/mL). This was expected from their lifestyles, and consistent with previous urban Japanese studies and other recent Asian studies in which it is reported that lighter skin tones are culturally preferred [14,15,31–33]. The thresholds for 25(OH)D levels (10 ng/mL for severe deficiency, and 20 ng/mL for deficiency) were derived from non-pregnant populations and an optimal serum level during pregnancy has not been established. However, it is at least suggested that Japanese pregnant women have lower vitamin D levels compared with a century ago, at which time the majority of the populations engaged in agriculture or fishery, spending many hours outside every day; among subjects who reported themselves being exposed to sunlight at least 15 minutes on more than 5 days a week without UV protection in neck and hands and consumed more than 7.0 **μ**g/day of dietary vitamin D, 49 of 50 subjects (98.0%) showed 25(OH)D above 10 ng/mL with the mean 25(OH)D level of 20.2 ng/mL throughout the year. Because vitamin D has functions in various organs, deficiency can cause or contribute to a variety of diseases [34]. The association between vitamin D deficiency and specific morbidities, especially diseases that is increasing in these decades, should be further investigated.

In this study, the average daily vitamin D intake among women with 25(OH)D levels above 20 ng/mL in winter and spring was 6.3 **μ**g/day, which is similar to reports in Norwegian pregnant women (7.0 **μ**g/day) [35]. Although the Japanese Guideline for Nutrition suggest 7.0 **μ**g/day, and this appears reasonable for Japanese pregnant women based on the results of our study, it is also important to note that 25(OH)D levels above 20 ng/mL were not achieved only by diet for majority of the women in winter and spring. This leads to the proposal that dietary intake of 7.0 **μ**g/day is necessary, but not sufficient to maintain adequate 25(OH)D levels at least 20 ng/mL in Japan.

Although vitamin D supplementation was reported by few pregnant women (5.1%) and most consumed only 100 to 200 IU/day of vitamin D in our study, the serum 25(OH)D levels increased by as much as 4 ng/mL in supplemented women. This figure is consistent with the previous report that showed 100IU of vitamin D increased the 25(OH)D level by 2 to 3 ng/mL in subjects with serum 25(OH)D levels of less than 15 ng/mL[34].

Darker skin is generally a risk factor for a low level of 25(OH)D[34]. However, in our study, comprising subjects of almost uniform ethnicity, the self-reported fair-colored skin had a tendency toward lower 25(OH)D levels. This subpopulation may have avoided sunlight to an extreme due to fear of skin cancer. Among Caucasians, especially those who emigrated to a low latitude area, UV exposure is a definite risk factor for skin cancer development. However, skin cancer mortality is very low in Japanese living in Japan, even in those who were children in the era without UV protection. The skin cancer mortality is 1.2 / 100 thousand Japanese women, while colon cancer mortality is 36.5 / 100 thousand Japanese women in 2017 [36]. The natural skin tone may be adapted to the sunlight in Japan as an evolutionary feature related to island dwelling. Skin production of vitamin D is thought to be accomplished after exposure to moderate sunlight for several (in summer) to several ten (in winter) minutes without causing burns [3,34]. National Institute for Environmental Studies comments that getting exposure to UV ray for several (in summer) to several ten (in winter) minutes in Japan that will never reach 1 MED (Minimal Erythema Dose) for people with skin photo type III (Japanese people), will lead vitamin D synthesis while minimizing its harms [37]. And it provides in real-time the amount of vitamin D synthesized in the body at some locations in Japan on the web [38], based on the logics described by Miyauchi and Nakajima[39]. Individuals should also be informed of the benefits of UV rays when alerted about its risk, with the available information shown above.

Major strengths of this study were a relatively large sample size based on a large population-based birth cohort (from the JECS), and a high response rate for various background questionnaires including both dietary intake of vitamin D and frequency of sunlight exposure, which will contribute to high generalizability. Despite the strengths, this study has some limitations. FFQ and frequency of sunlight exposure were both self-reported, and there may be some mis-categorizations. A uniform questionnaire was used for sunlight exposure throughout a year, which may not be a meaningful measure of differences in sunlight exposure in winter, when it is estimated that at least several ten minutes of sunlight is necessary for vitamin D production in skin, whereas the questionnaire asked the frequency of “at least 15 minute exposure /day” per week. Finally, the present results are applicable to only Japanese pregnant women, as it is known that 25(OH)D levels differ between ethnicities[34].

In conclusion, vitamin D deficiency is very severe in Japanese pregnant women, and lifestyle factors including the frequency of sunlight exposure and dietary intake of vitamin D have a clinically relevant impact on serum levels. This suggests that vitamin D level may be enhanced by changes in lifestyle. Pregnant women should be informed of both the risks and benefits of UV ray. Further investigations are required to establish the impact of vitamin D deficiency on morbidities.

## References

[1] Holick MF, Chen TC. Vitamin D deficiency: a worldwide problem with health consequences. The American journal of clinical nutrition. 2008;87(4):1080s–6s. Epub 2008/04/11. 10.1093/ajcn/87.4.1080S . 18400738

[2] Baggerly CA, Cuomo RE, French CB, Garland CF, Gorham ED, Grant WB, et al Sunlight and Vitamin D: Necessary for Public Health. Journal of the American College of Nutrition. 2015;34(4):359–65. Epub 2015/06/23. 10.1080/07315724.2015.1039866 26098394 PMC4536937

[3] Holick MF. Vitamin D deficiency. The New England journal of medicine. 2007;357(3):266–81. Epub 2007/07/20. 10.1056/NEJMra070553 . 17634462

[4] Barebring L, Bullarbo M, Glantz A, Leu Agelii M, Jagner A, Ellis J, et al Preeclampsia and Blood Pressure Trajectory during Pregnancy in Relation to Vitamin D Status. PloS one. 2016;11(3):e0152198 Epub 2016/03/31. 10.1371/journal.pone.0152198 27022948 PMC4811441

[5] Chakhtoura M, Nassar A, Arabi A, Cooper C, Harvey N, Mahfoud Z, et al Effect of vitamin D replacement on maternal and neonatal outcomes: a randomised controlled trial in pregnant women with hypovitaminosis D. A protocol. BMJ open. 2016;6(3):e010818 Epub 2016/03/10. 10.1136/bmjopen-2015-010818 26956166 PMC4785305

[6] Hossein-nezhad A, Holick MF. Optimize dietary intake of vitamin D: an epigenetic perspective. Current opinion in clinical nutrition and metabolic care. 2012;15(6):567–79. Epub 2012/10/19. 10.1097/MCO.0b013e3283594978 . 23075936

[7] Thorne-Lyman A, Fawzi WW. Vitamin D during pregnancy and maternal, neonatal and infant health outcomes: a systematic review and meta-analysis. Paediatric and perinatal epidemiology. 2012;26 Suppl 1:75–90. Epub 2012/07/07. 10.1111/j.1365-3016.2012.01283.x 22742603 PMC3843348

[8] Tanaka K, Hitsumoto S, Miyake Y, Okubo H, Sasaki S, Miyatake N, et al Higher vitamin D intake during pregnancy is associated with reduced risk of dental caries in young Japanese children. Annals of epidemiology. 2015;25(8):620–5. Epub 2015/05/10. 10.1016/j.annepidem.2015.03.020 . 25956333

[9] Hamazaki K, Natori T, Kurihara S, Murata N, Cui ZG, Kigawa M, et al Fish consumption and depressive symptoms in undergraduate students: A cross-sectional analysis. European psychiatry : the journal of the Association of European Psychiatrists. 2015;30(8):983–7. Epub 2015/10/27. 10.1016/j.eurpsy.2015.09.010 . 26495907

[10] Ministry of Health LaW. National Health and Nutrition Survey 2003 [cited 2016 Apr. 20]. Available from: http://www.mhlw.go.jp/bunya/kenkou/eiyou-chosa2-01/index.html,.

[11] Ministry of Health LaW. National Health and Nutrition Survey. 2012.

[12] Otsuka R, Yatsuya H, Tamakoshi K. Descriptive epidemiological study of food intake among Japanese adults: analyses by age, time and birth cohort model. BMC public health. 2014;14:328 Epub 2014/04/10. 10.1186/1471-2458-14-328 24712924 PMC3997235

[13] Hayashi F, Takimoto H, Yoshita K, Yoshiike N. Perceived body size and desire for thinness of young Japanese women: a population-based survey. The British journal of nutrition. 2006;96(6):1154–62. Epub 2006/12/22. . 17181892 10.1017/bjn20061921

[14] Shibata M, Suzuki A, Sekiya T, Sekiguchi S, Asano S, Udagawa Y, et al High prevalence of hypovitaminosis D in pregnant Japanese women with threatened premature delivery. Journal of bone and mineral metabolism. 2011;29(5):615–20. Epub 2011/03/09. 10.1007/s00774-011-0264-x . 21384110

[15] Shiraishi M, Haruna M, Matsuzaki M, Murayama R. Demographic and lifestyle factors associated with vitamin D status in pregnant Japanese women. Journal of nutritional science and vitaminology. 2014;60(6):420–8. Epub 2014/01/01. 10.3177/jnsv.60.420 . 25866306

[16] Kanatani KT, Adachi Y, Sugimoto N, Noma H, Onishi K, Hamazaki K, et al Birth cohort study on the effects of desert dust exposure on children's health: protocol of an adjunct study of the Japan Environment & Children's Study. BMJ open. 2014;4(6):e004863 Epub 2014/06/25. 10.1136/bmjopen-2014-004863 24958210 PMC4067890

[17] Kawamoto T, Nitta H, Murata K, Toda E, Tsukamoto N, Hasegawa M, et al Rationale and study design of the Japan environment and children's study (JECS). BMC public health. 2014;14:25 Epub 2014/01/15. 10.1186/1471-2458-14-25 24410977 PMC3893509

[18] Nitta H. Outline of the Japan Environment and Children's Study and the Framework of Genome Analysis. Nihon eiseigaku zasshi Japanese journal of hygiene. 2016;71(1):91–3. Epub 2016/02/03. 10.1265/jjh.71.91 . 26832622

[19] Hollis BW, Kamerud JQ, Selvaag SR, Lorenz JD, Napoli JL. Determination of vitamin D status by radioimmunoassay with an 125I-labeled tracer. Clinical chemistry. 1993;39(3):529–33. Epub 1993/03/01. . 8448871

[20] Litonjua AA, Carey VJ, Laranjo N, Harshfield BJ, McElrath TF, O'Connor GT, et al Effect of Prenatal Supplementation With Vitamin D on Asthma or Recurrent Wheezing in Offspring by Age 3 Years: The VDAART Randomized Clinical Trial. Jama. 2016;315(4):362–70. Epub 2016/01/28. 10.1001/jama.2015.18589 . 26813209 PMC7479967

[21] Ishihara J, Sobue T, Yamamoto S, Yoshimi I, Sasaki S, Kobayashi M, et al Validity and reproducibility of a self-administered food frequency questionnaire in the JPHC Study Cohort II: study design, participant profile and results in comparison with Cohort I. Journal of epidemiology / Japan Epidemiological Association. 2003;13(1 Suppl):S134–47. Epub 2003/04/19. . 12701641 10.2188/jea.13.1sup_134 PMC9767691

[22] Ogawa K, Tsubono Y, Nishino Y, Watanabe Y, Ohkubo T, Watanabe T, et al Validation of a food-frequency questionnaire for cohort studies in rural Japan. Public health nutrition. 2003;6(2):147–57. Epub 2003/04/05. 10.1079/PHN2002411 . 12675957

[23] Wakai K. A review of food frequency questionnaires developed and validated in Japan. Journal of epidemiology / Japan Epidemiological Association. 2009;19(1):1–11. Epub 2009/01/24. 10.2188/jea.JE20081007 19164867 PMC3924089

[24] Sasaki S, Takahashi T, Iitoi Y, Iwase Y, Kobayashi M, Ishihara J, et al Food and nutrient intakes assessed with dietary records for the validation study of a self-administered food frequency questionnaire in JPHC Study Cohort I. Journal of epidemiology / Japan Epidemiological Association. 2003;13(1 Suppl):S23–50. Epub 2003/04/19. . 12701630 10.2188/jea.13.1sup_23 PMC9767698

[25] Communications MoIAa. Japan Statistical Yearbook 2013. First Editorial Section, Statistical Library, Statistical Research and Training Institute 2013 [cited 2016 Apr. 19]. Available from: http://www.stat.go.jp/data/nihon/18.htm.

[26] Ministry of Health LaW. The national health and nutrition survey in Japan, 2011. 2011 [cited 2016 Apr.19]. Available from: http://www.mhlw.go.jp/bunya/kenkou/eiyou/dl/h24-houkoku-06.pdf.

[27] School Health Survey. Available from: http://www.e-stat.go.jp/SG1/estat/eStatTopPortalE.do.

[28] Statistics Bureau MoIA, and Communications. The Population and Households of Japan 2010. Available from: http://www.stat.go.jp/data/kokusei/2010/pdf/waga3.pdf.

[29] Ministry of Health LaW. Overview of Dietary Reference Intakes for Japanese [cited 2016 May 1st]. Available from: http://www.mhlw.go.jp/bunya/kenkou/syokuji_kijyun.html, http://www.mhlw.go.jp/stf/seisakunitsuite/bunya/0000061795.html.

[30] Hasegawa A, Usui C, Kawano H, Sakamoto S, Higuchi M. Characteristics of body composition and resting energy expenditure in lean young women. Journal of nutritional science and vitaminology. 2011;57(1):74–9. Epub 2011/04/23. . 21512294 10.3177/jnsv.57.74

[31] Choi R, Kim S, Yoo H, Cho YY, Kim SW, Chung JH, et al High prevalence of vitamin D deficiency in pregnant Korean women: the first trimester and the winter season as risk factors for vitamin D deficiency. Nutrients. 2015;7(5):3427–48. Epub 2015/05/15. 10.3390/nu7053427 25970148 PMC4446760

[32] Xiao JP, Zang J, Pei JJ, Xu F, Zhu Y, Liao XP. Low maternal vitamin D status during the second trimester of pregnancy: a cross-sectional study in Wuxi, China. PloS one. 2015;10(2):e0117748 Epub 2015/02/07. 10.1371/journal.pone.0117748 25659105 PMC4320063

[33] Pratumvinit B, Wongkrajang P, Wataganara T, Hanyongyuth S, Nimmannit A, Chatsiricharoenkul S, et al Maternal Vitamin D Status and Its Related Factors in Pregnant Women in Bangkok, Thailand. PloS one. 2015;10(7):e0131126 Epub 2015/07/07. 10.1371/journal.pone.0131126 26147381 PMC4492949

[34] Hossein-nezhad A, Holick MF. Vitamin D for health: a global perspective. Mayo Clin Proc. 2013;88(7):720–55. 10.1016/j.mayocp.2013.05.011 23790560 PMC3761874

[35] Henriksen C, Brunvand L, Stoltenberg C, Trygg K, Haug E, Pedersen JI. Diet and vitamin D status among pregnant Pakistani women in Oslo. European journal of clinical nutrition. 1995;49(3):211–8. Epub 1995/03/01. . 7774537

[36] Cancer Registry and Statistics. Cancer Information Service, National Cancer Center, Japan. [cited 2019 Feb 4th]. Available from: https://ganjoho.jp/reg_stat/statistics/dl/index.html#mortality

[37] Information on Vitamin D synsthesis/Erythermal UV. National Institute for Environmental Studies. [cited 2019 Feb 4th]. Available from: http://db.cger.nies.go.jp/dataset/uv_vitaminD/en/remarks.html

[38] Information on Vitamin D synsthesis/Erythermal UV. National Institute for Environmental Studies. [cited 2019 Feb 4th]. Available from: http://db.cger.nies.go.jp/dataset/uv_vitaminD/en/radiation.html

[39] Miyauchi M. and Nakajima H. Determining an effective UV radiation exposure time for vitamin D synthesis in the skin without risk to health: Simplified estimations from UV observation. Photochem. Photobiol. 2016;92:863–9. 10.1111/php.12651 27754554

